# Microstructure Informed Tractography: Pitfalls and Open Challenges

**DOI:** 10.3389/fnins.2016.00247

**Published:** 2016-06-06

**Authors:** Alessandro Daducci, Alessandro Dal Palú, Maxime Descoteaux, Jean-Philippe Thiran

**Affiliations:** ^1^Signal Processing Lab, Electrical Engineering, École Polytechnique Fédérale de LausanneLausanne, Switzerland; ^2^Radiology Department, University Hospital CenterLausanne, Switzerland; ^3^Sherbrooke Connectivity Imaging Lab, Computer Science, Université de SherbrookeSherbrooke, QC, Canada; ^4^Mathematics and Computer Science Department, University of ParmaParma, Italy

**Keywords:** diffusion MRI, tractography, microstructure imaging, interpretation, pitfalls, open challenges

## Abstract

One of the major limitations of diffusion MRI tractography is that the fiber tracts recovered by existing algorithms are not truly quantitative. Local techniques for estimating more quantitative features of the tissue microstructure exist, but their combination with tractography has always been considered intractable. Recent advances in local and global modeling made it possible to fill this gap and a number of promising techniques for *microstructure informed tractography* have been suggested, opening new and exciting perspectives for the quantification of brain connectivity. The ease-of-use of the proposed solutions made it very attractive for researchers to include such advanced methods in their analyses; however, this apparent simplicity should not hide some critical open questions raised by the complexity of these very high-dimensional problems, otherwise some fundamental issues may be pushed into the background. *The aim of this article is to raise awareness* in the diffusion MRI community, notably researchers working on brain connectivity, about some potential pitfalls and modeling choices that make the *interpretation of the outcomes from these novel techniques rather cumbersome*. Through a series of experiments on synthetic and real data, we illustrate practical situations where erroneous and severely biased conclusions may be drawn about the connectivity if these pitfalls are overlooked, like the presence of partial/missing/duplicate fibers or the critical importance of the diffusion model adopted. Microstructure informed tractography is a young but very promising technology, and by acknowledging its current limitations as done in this paper, we hope our observations will trigger further research in this direction and new ideas for truly quantitative and biologically meaningful analyses of the connectivity.

## 1. Introduction

It is commonly acknowledged that the human brain is the most complex system in nature. The presence of pathological conditions in its intricate structure may lead to a wide variety of neurological disorders and, thus, the availability of tools to investigate its organization is of paramount importance. *Diffusion Magnetic Resonance Imaging* (dMRI) is one of such tools that allows *in-vivo* and non-invasive investigation of brain connectivity. In biological tissues, the natural motion of water molecules is highly influenced by the microstructural environment and, in the white matter, the anisotropy of the resulting random process can be exploited to probe important features of the neuronal tissue (Le Bihan et al., 1986; Beaulieu, 2002).

**Figure 1:**
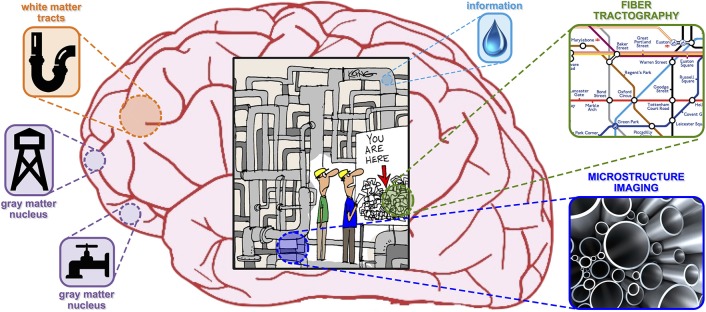
**Plumbing metaphor picturing *microstructure informed tractography* as a tool to evaluate the health condition of the water supply network of a big city**.

To help the general reader who is unfamiliar with the field, the metaphor illustrated in Figure 1 might be convenient. One can imagine brain imaging as a tool to assess the health condition of the water supply network of a big city, in which the treated water (i.e., the information) is distributed to the consumers (i.e., gray matter nuclei) through a very intricate pipe network (i.e., white matter nerves). To analyze the state of the system, the plumber has at his disposal a powerful toolbox (i.e., dMRI) which allows him to perform two complementary evaluations. On one hand, the *topology* of the network can be assessed using *tractography*; for an overview, see Mangin et al. (2013) and references therein. However, in spite of the high number of algorithms developed, none of the existing techniques can actually measure the capacity of the pipes (i.e., the amount of water that can flow through them). To obtain this information, the plumber can use another tool called *microstructure imaging*; for a review, see Panagiotaki et al. (2012) and references therein. These methods can characterize the *morphology* of the pipes in each district but, on the other hand, they cannot establish the origin or the consumers of the water passing through them.

*Microstructure informed tractography* is a relatively new area of research that, translated into the previous figure of speech, aims at combining these two pieces of information using global optimization techniques in order to draw a quantitative map of the pipe network which, today, is not available. In fact, several orders of magnitude separate the resolution achievable with dMRI from the actual size of the axons and each reconstructed trajectory has to be considered as representative of a coherent set of real anatomical fibers, the amount of which is not easy to assess. As a consequence, nowadays the structural connectivity between brain regions is quantified by counting the number of recovered tracts or averaging a scalar map along them, e.g., Fractional Anisotropy (FA); either way, these quantities are only indirectly related to the actual underlying neuronal connectivity (Jones et al., 2012). In the past few years, this limitation has received a fast-growing interest in the field and a number of interesting solutions have been proposed. A first class of methods (Kreher et al., 2008; Fillard et al., 2009; Reisert et al., 2011, 2014; Christiaens et al., 2015; Girard et al., 2015) reconstruct the full tractogram, i.e., set of fiber tracts, from the measured data in a *bottom-up* fashion. The tracts are formed starting from a collection of short segments, whose signal contribution in each voxel is defined using tissue models, that are encouraged to interact and form long chains using global energy minimization. Conversely, *top-down* approaches start from a collection of tracts constructed using standard tractography methods, and attempt to assess their actual contribution, or some other features of interest like the average axon diameter, resorting to various optimization techniques such as stochastic algorithms (Sherbondy et al., 2009, 2010), non-linear gradient descent (Smith et al., 2013, 2015), random-walk simulations (Lemkaddem et al., 2014) or, more recently, convex optimization (Daducci et al., 2013, 2014a; Pestilli et al., 2014). Despite using quite different strategies, all previous solutions share the same goal: *combine tractography with tissue microstructural models* in the pursuit of more quantitative and biologically oriented estimation of brain connectivity.

Recent advances in software development^1^ turned the use of such complex models into a rather straightforward operation but, on the other hand, this ease-of-use also pushed some fundamental issues into the background, e.g., the critical importance of the diffusion model adopted or the impact on the results of partial/missing/duplicate fibers. The *interpretation of the outcomes* from such *high-dimensional problems* represents a nontrivial task and it is subject to a number of potential pitfalls that might be not so obvious at first glance. If overlooked and these tools are used as black boxes, erroneous inferences might be drawn from the data, leading to very deceptive conclusions about the connectivity in the brain. The *purpose of this article is to raise awareness* of the reader about some subtle pitfalls that may be hidden by the apparent simplicity of these novel techniques, but that can severely impact on the interpretation of the results. Most of the issues we discuss are not just concerns in the context of microstructure informed tractography but, in general, are relevant to most of the existing tractography algorithms, and can potentially bias any connectivity analysis with them. Advantages and limitations of classical tractography have been extensively reviewed and discussed in several previous studies, for example Jones (2010), Jbabdi and Johansen-Berg (2011), Fillard et al. (2011), Côté et al. (2013), Thomas et al. (2014), and Jbabdi et al. (2015). Therefore we hope this article will serve as a complement to the existing literature and will help potential users of these novel techniques to *correctly interpret their results* and, also, give ideas to the methods developers about the current open challenges that still need to be solved, hence triggering further research in this direction.

The manuscript is organized as follows. First, we call attention to the close affinity that exists between microstructure informed tractography methods, notably the linear formulation recently introduced by Daducci et al. (2013, 2014a), and classical dictionary-based techniques for recovering the local fiber structure in a voxel. This analogy will provide us with a solid mathematical framework to describe strengths and pitfalls of global reconstruction methods that are inherited from their local counterpart, while keeping the presentation simple and easy to follow. In the remainder of the article, we perform a series of experiments on synthetic and real data to illustrate some of the situations where the interpretation of the outcomes might go terribly wrong if these pitfalls are overlooked, describing the causes and providing simple but clear explanations. Please note that although existing methods vary considerably in terms of approach and assumptions, e.g., bottom-up vs. top-down, most of the issues covered in this article are common to most of them or can be easily generalized.

## 2. A parallel with local reconstruction and inherited issues

With *local reconstruction* we refer to the branch of dMRI that deals with the estimation of the intra-voxel fiber structure from the acquired MR data. As known in the field, many features of interest, such as the fiber orientation distribution function (ODF) (Ramirez-Manzanares et al., 2007; Tournier et al., 2007) or more detailed microstructural properties of the tissue (Alexander et al., 2010; Daducci et al., 2015), can be expressed as linear combinations of given basis-functions, also called atoms, as follows:

(1) $$\begin{array}{l} \text{y}=\text{A}\text{x}+\eta , \end{array}$$

where $\text{y}\in {\mathbb{R}}_{+}^{N_{d}}$ is the vector containing the dMRI signal acquired in the voxel, η accounts for the acquisition noise, $\text{A}=\left\{a_{ij}\right\}\in {\mathbb{R}}^{N_{d}\times N_{k}}$ is the linear operator, or dictionary, that explicitly maps the feature of interest to the measurements through the *N*_*k*_ basis functions and $\text{x}\in {\mathbb{R}}_{+}^{N_{k}}$ are the corresponding contributions. These latter can be efficiently estimated, for instance, by solving the following general regularized least-squares problem:

!!

where || · ||_2_ is the standard ℓ_2_-norm in ℝ^*n*^, Φ(·) represents a generic function and λ ≥ 0 controls the relative strength of the regularization (Descoteaux et al., 2007).

**Figure 2:**
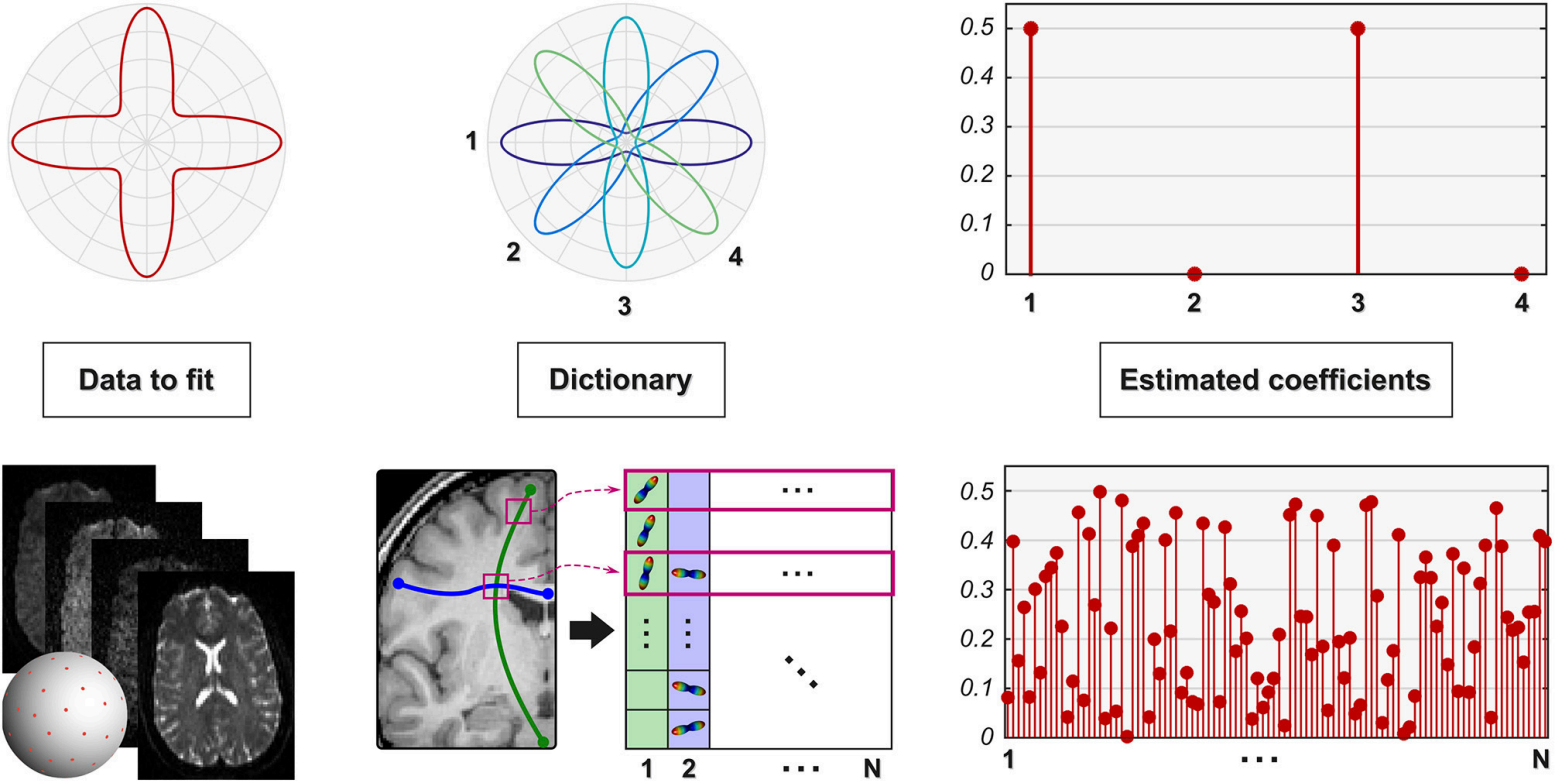
**Parallel between local and global reconstruction**. *Local reconstruction* methods usually recover the fiber configuration in a voxel by expressing the quantity of interest, e.g., ODF, as a linear combination of a given set of response functions (shown here in 2D for simplicity). Daducci et al. (2013, 2014a) showed that also microstructure informed tractography can be expressed using the same formulation where, instead of a single voxel, the dictionary models the whole dMRI image as a superimposition of the signal arising from all the fibers in a tractogram.

Daducci et al. (2013) showed that also the *microstructure informed tractography* problem can be recast in terms of the same linear formulation given in Equations (1) and (2); this framework was later generalized (Daducci et al., 2014a) to allow the combination of tractography with any microstructural tissue-model (Panagiotaki et al., 2012). For the aims of this study, the possibility to *use the same framework* to express both local and global problems allows us to describe known issues of local formulations (that have been extensively studied in the literature) and to readily extrapolate them to global approaches. This analogy is depicted in Figure 2. In the case of local reconstruction (top row), the estimation of the fiber configuration in each voxel is usually performed on a voxel-by-voxel basis or considering small neighborhoods. The dictionary reflects the specific features of interest and the data to fit; for instance, it may consist of Gaussian profiles as in Ramirez-Manzanares et al. (2007) and the estimated coefficients **x** correspond then to the contributions of the fiber populations present in the voxel along any direction. In contrast, rather than fitting one voxel at the time, microstructure informed tractography methods (bottom row) consider simultaneously all the voxels of the brain using global optimization techniques. The dictionary consists in this case of a combination of the fiber tracts present in the tractogram with tissue forward-models to assess their contribution to the dMRI image along their trajectories; the interested reader is referred to Daducci et al. (2014a) for more details.

A variety of modeling approaches has been used by different optimization strategies to predict the contribution of the tracts in each imaging voxel, from the classical mixture of tensors (Reisert et al., 2011; Pestilli et al., 2014) to more sophisticated models that directly relate to features of the tissue microstructure (Sherbondy et al., 2010; Daducci et al., 2014a). For the scope of our analysis and sake of simplicity, in our experiments we adopted the Stick-Zeppelin-Ball model (Panagiotaki et al., 2012): axons represented as cylinders with zero radius, extra-cellular space modeled with anisotropic tensors and isotropic free diffusion. The estimated coefficients **x** correspond to the actual weight (or volume) of each fiber, possibly in addition to other signal contaminations from non-fiber tissues, e.g., cerebrospinal fluid (CSF). Please note that more realistic biophysical models could be easily considered in this framework, e.g., to account for bundles composed of axon populations with different radii as in Sherbondy et al. (2010) and Daducci et al. (2014a), but their use would only complicate the exposition without affecting the observations discussed in this work. In Section 3, though, we will examine the impact on the recovered fiber parameters of small variations to this tissue model.

### 2.1. Data and experiments

To highlight the hidden dangers mentioned before in interpreting the outcomes, we performed a series of experiments on both synthetic and real data aimed at evaluating microstructure informed tractography methods in different conditions. In particular, we first illustrate some well-known issues of local methods with the help of a *simple synthetic example*, for which the expected behavior is easily explained; then we exploit the parallel between local and global reconstruction to extrapolate these observations to global approaches. The phantom consists of a single voxel with two fiber populations crossing at 90° (Figure 2, top-left). For the sake of illustration, both the signal corresponding to this configuration and the response functions of the dictionary have been simulated using the classical multi-tensor model (Tuch et al., 2002); all the observations in this article remain the same if more complex generative models are used (Soderman and Jonsson, 1995; Assaf and Basser, 2005). Also, although 2D ODF are shown in the plots, the actual experiments were performed in signal space on the sphere. Global reconstruction was tested on *real data* that is publicly-available; specifically, we used the same two-shell dataset from Daducci et al. (2014a), i.e., 24 images at *b* = 700s/mm^2^ and 48 at *b* = 2000s/mm^2^, as well as 10 datasets from the Human Connectome Project (HCP) (Van Essen et al., 2012), i.e., 90 measurements at *b* = 2000s/mm^2^ as done in Pestilli et al. (2014). If not otherwise specified, the two-shell dataset was used in all real-data experiments.

For the scope of this paper, tractography was performed with the probabilistic iFOD2 algorithm (Tournier et al., 2012) and the GIBBS tracker (Reisert et al., 2011), using default parameters. The corresponding dictionaries were created by combining the streamlines in each tractogram with the Stick-Zeppelin-Ball model, implemented in the DIPY library (Garyfallidis et al., 2014), and assuming diffusivities values typical for *in-vivo* human data, as in Alexander et al. (2010), Zhang et al. (2012), and Daducci et al. (2014a): longitudinal *d*_∥_ = 1.7 × 10^−3^mm^2^ ∕ s, perpendicular *d*_⊥_ = 0.5 × 10^−3^mm^2^ ∕ s and same *d*_∥_ in the extra-cellular space, and two isotropic compartments with *d* ∈ {1.7, 3.0} × 10^−3^ mm^2^ ∕ s. For more details on the construction of the dictionary, please refer to Daducci et al. (2014a). All experiments were carried out using the publicly-available^2^ COMMIT framework, adopting the Douglas-Rachford algorithm (Combettes and Pesquet, 2007) with no regularization (λ = 0) to solve Equation (2) for both local and global problems. This data and experimental setup was used consistently throughout the manuscript.

### 2.2. Missing atoms/fibers

**Figure 3:**
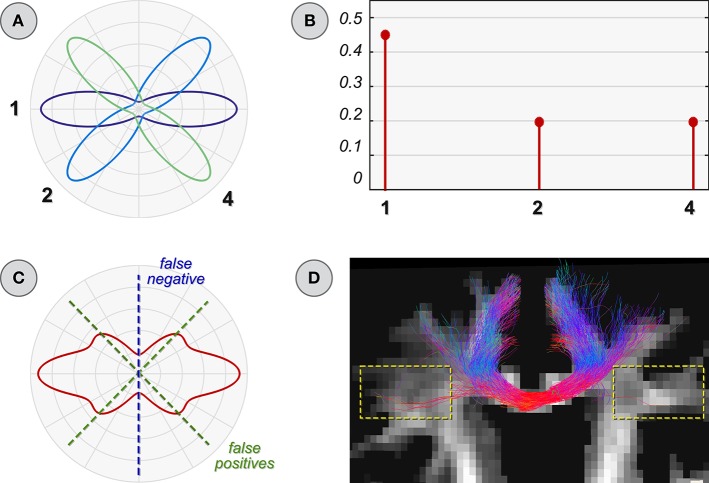
**Improper dictionary: missing atoms**. **(A)** Schematic illustration of a dictionary with a missing atom (number 3 in Figure 2). Estimated coefficients **(B)** and ODF **(C)** when using it to reconstruct the voxel configuration in Figure 2. **(D)** Parallel with global reconstruction: the common difficulty of classical tractography algorithms in tracking the callosal projection fibers (yellow boxes) is a typical scenario that leads to missing atoms (i.e., fibers) in the dictionary.

Figure 3 illustrates the situation where some *atoms are missing in the dictionary*. It is straightforward to realize how the 90° configuration (top-left in Figure 2) cannot be accurately described in terms of only atoms 1, 2, and 4. The best fit actually consists in assigning a weight of 0.5 to atoms 1 and 3; however, as this latter is not present in the dictionary (Figure 3A), the optimization compensates for this deficiency by assigning a contribution to atoms 2 and 4 instead (Figure 3B). The consequences are clearly visible in Figure 3C: not only the vertical bundle corresponding to atom 3 is obviously not recovered, i.e., *false negative*, but two spurious fiber populations might also be incorrectly detected, i.e., *false positives*.

In the context of global reconstruction, this situation arises when a tracking algorithm fails to reconstruct some fibers, which are thus not accounted for in the dictionary. A typical example, as shown in Reisert et al. (2011) and Mangin et al. (2013), is the well-known difficulty of classical streamline tractography in reconstructing the lateral projections of the corpus callosum (Figure 3D). Besides clearly ignoring these fibers in the analysis, global methods might also *incorrectly assign a contribution to other bundles* and thus infer that two gray matter regions are actually connected when, perhaps, these fibers may not exist at all. Therefore, missing fibers, or in the specific context of this example missing atoms in the dictionary, can be hugely problematic for the correct assessment of the fiber contributions. The construction of an adequate set of fiber tracts, either by top-down or bottom-up approaches, is therefore of utmost importance and currently an open problem in tractography, in particular for those methods which attempt to estimate the contribution of the tracts to the measured data or to a feature of interest (e.g., ODF).

### 2.3. Duplicate atoms/fibers

**Figure 4:**
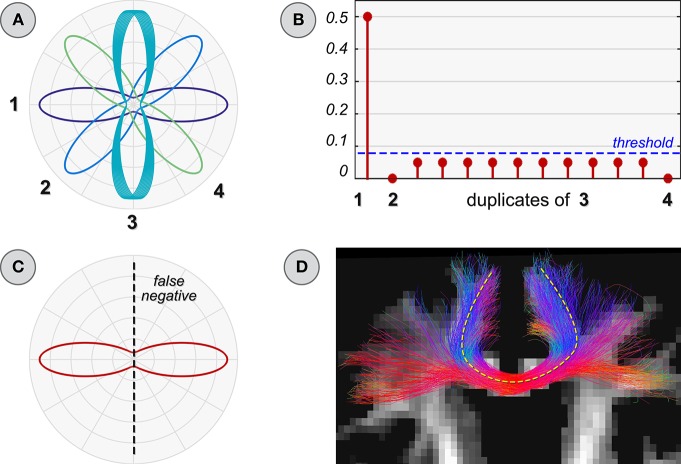
**Improper dictionary: duplicate atoms**. **(A)** Dictionary with replicas of an atom (number 3 in Figure 2). Estimated coefficients **(B)** and ODF **(C)** when using it to reconstruct the voxel configuration in Figure 2. **(D)** Parallel with global reconstruction: the trajectories of an easy-to-track bundle (yellow line) can be reconstructed many times in a tractogram and the corresponding atoms can be assigned very small contributions; this could lead to conclude that each duplicate fiber is spurious.

The most straightforward solution one can think of for ensuring the inclusion of all possible fibers is to *merge tractograms from different algorithms*. However, if performed without due care, this operation can generate another subtle issue. For example, the dictionary in Figure 4A contains all the response functions that are required to fit correctly the 90° configuration in the top-left of Figure 2, but it also includes some duplicates. Depending on the algorithm used to solve Equation (2), the actual contribution of a response function could be arbitrarily distributed among its copies; the higher this number and the more the corresponding coefficients tend to be small (Figure 4B). In this case, however, a low weight does not necessarily mean that those atoms are less important than others in explaining the data. Besides, it is worth recalling that local methods usually employ a threshold to determine when a response function can be considered spurious (Tournier et al., 2007); in presence of duplicates, these atoms are more likely to be discarded because their coefficients can fall below this level, and thus *false negatives* can be generated (Figure 4C). Actually, the same issue may arise also without such threshold if a form of regularization is naively applied. As an example, using Φ(*x*) = ||*x*||_1_ for promoting sparsity (Candès et al., 2006; Donoho, 2006) in the tractogram, small coefficients may tend to be suppressed depending on how the specific algorithm for solving Equation (2) handles highly correlated atoms. Thus, false negatives may be generated also in this case and different regularization functions may lead to even more unpredictable results; this is why we adopted classical least squares in all our experiments.

Major fiber bundles such as the U-shaped callosal radiations, the corticospinal tract or the inferior fronto-occipital fasciculus are easily reconstructed by most tractography algorithms and they are likely overrepresented in a tractogram. Figure 4D shows a subset of the tracts after merging the tractogram of Figure 3D with the output of the GIBBS tracker, which now include the callosal projections that were missed previously. It can be noticed that the callosal radiations appear denser than before, as indeed these fibers were easily reconstructed by both algorithms; hence the corresponding atoms in the dictionary are likely to be assigned small weights. As a consequence, if the *raw estimated weights are used to discriminate between true and spurious fibers*, these might be incorrectly labeled as spurious simply because they are so easy to track and many replicas are actually recovered in the tractogram. Note that, once again, this potential danger is not an issue only for dictionary-based approaches but it may arise also in other techniques, both top-down and bottom-up, because in presence of duplicates there exist infinite solutions providing the same data fitting but characterized by an arbitrary distribution of the actual fiber weight among its copies, which depends on the specific optimization algorithm.

### 2.4. Partial fibers

**Figure 5:**
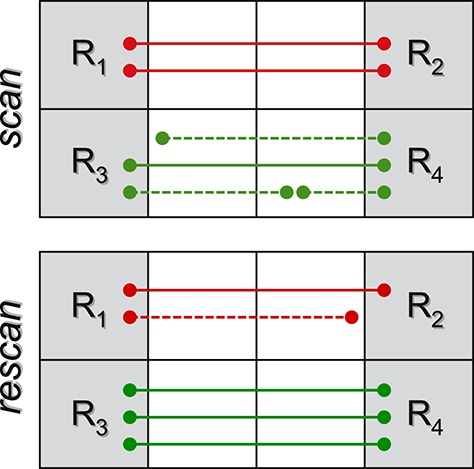
**Toy problem to illustrate some issues of partial fibers on connectivity analysis**. Tracts connecting the four gray-matter regions *R*_1_, …, *R*_4_ are marked as solid lines and those stopping prematurely in the white matter as dashed. It is well-known that the (random) number of partial fibers recovered with tractography can significantly bias the estimation; unfortunately, if these partial fibers are not properly handled, also microstructure informed tractography techniques can result ineffective.

Despite the fact that axons connect neurons located in the gray matter, a number of factors actually cause part of the tracts reconstructed with tractography to *stop prematurely in the white matter*; it was shown in Côté et al. (2013) and Girard et al. (2014) that up to 70% of the streamlines produced with state-of-the-art tracking algorithms actually do not reach the gray matter. This is a very well-known problem in tractography which, in turn, can severely bias any subsequent connectivity analysis. Probabilistic algorithms (Behrens et al., 2003; Parker et al., 2003) have been proposed to deal with this uncertainty, but the interpretation of the generated probabilistic maps as connection strength is a controversial matter (Jones et al., 2012). In this section we show that, if these “partial fibers” are not properly considered, also microstructure informed tractography techniques can result ineffective in fixing this issue with estimating the connectivity. As an example, consider the simplistic scan-rescan analysis in Figure 5 and assume it corresponds to a healthy subject, i.e., no significant change is expected. If the connection strength between the four regions *R*_1_, …, *R*_4_ is quantified by the fiber count (Jones et al., 2012), then a reduction in *R*_1_ ↔ *R*_2_ connectivity is observed between the two sessions (from 2 to 1), and an increase in *R*_3_ ↔ *R*_4_ (from 1 to 3), as the dashed tracts do not connect and so do not contribute to the count. Hence, one might erroneously infer that some sort of “disruption” took place in the former case and “axonal remodeling” in the latter.

Microstructure informed tractography methods may help obtaining unbiased connectivity estimates, but only in case these partial fibers are removed from the tractogram before optimization. In fact, their presence can potentially lead to the same problem discussed in the previous section, i.e., contribution of a bundle distributed among similar tracts; besides, although partial fibers do not connect, they still contribute to the dMRI image during optimization. As a consequence, they “steal” in practice part of the actual weight of a bundle and bias the final connectivity estimation. It is interesting to notice that, since the tracts are almost the same between scan and re-scan sessions, the two tractograms actually fit the data equally well and thus, in such situations, it is very difficult to spot possible troubles from the fitting errors. In contrast, if global optimization is run using only the tracts that actually connect, the total contribution of each bundle can be recovered consistently between the two sessions, as the trajectories provide a valid support for the optimization; this is true also for bottom-up approaches. In the example shown in Figure 5, the weight of the connection *R*_1_ ↔ *R*_2_ will be estimated to be 1 in both cases: in the first scan it is distributed over the two tracts (but with sum equal to 1, as the total contribution has to fit the measured data) whereas, in the re-scan, the sole fiber that reached the gray matter is assigned a weight of 1. Similarly for *R*_3_ ↔ *R*_4_.

## 3. Importance of the forward model

The tissue forward-model is a very important ingredient for investigating the evidence underpinning tractograms. Of course, it is well-known that simpler models usually result in higher fitting errors, but it is also easy to realize that when a model does not explain adequately the measured signal, then the estimated parameters are most likely unreliable and biased inferences may be drawn about the connectivity. Although the identification of the most appropriate model is an ongoing quest in the field, and beyond the scope of this work, in this section we wish to draw the attention of the reader to the *repercussions of small differences in the tissue model on the recovered parameters* about the tractogram and, consequently, on the estimation of the connectivity.

### 3.1. Fit accuracy

**Figure 6:**
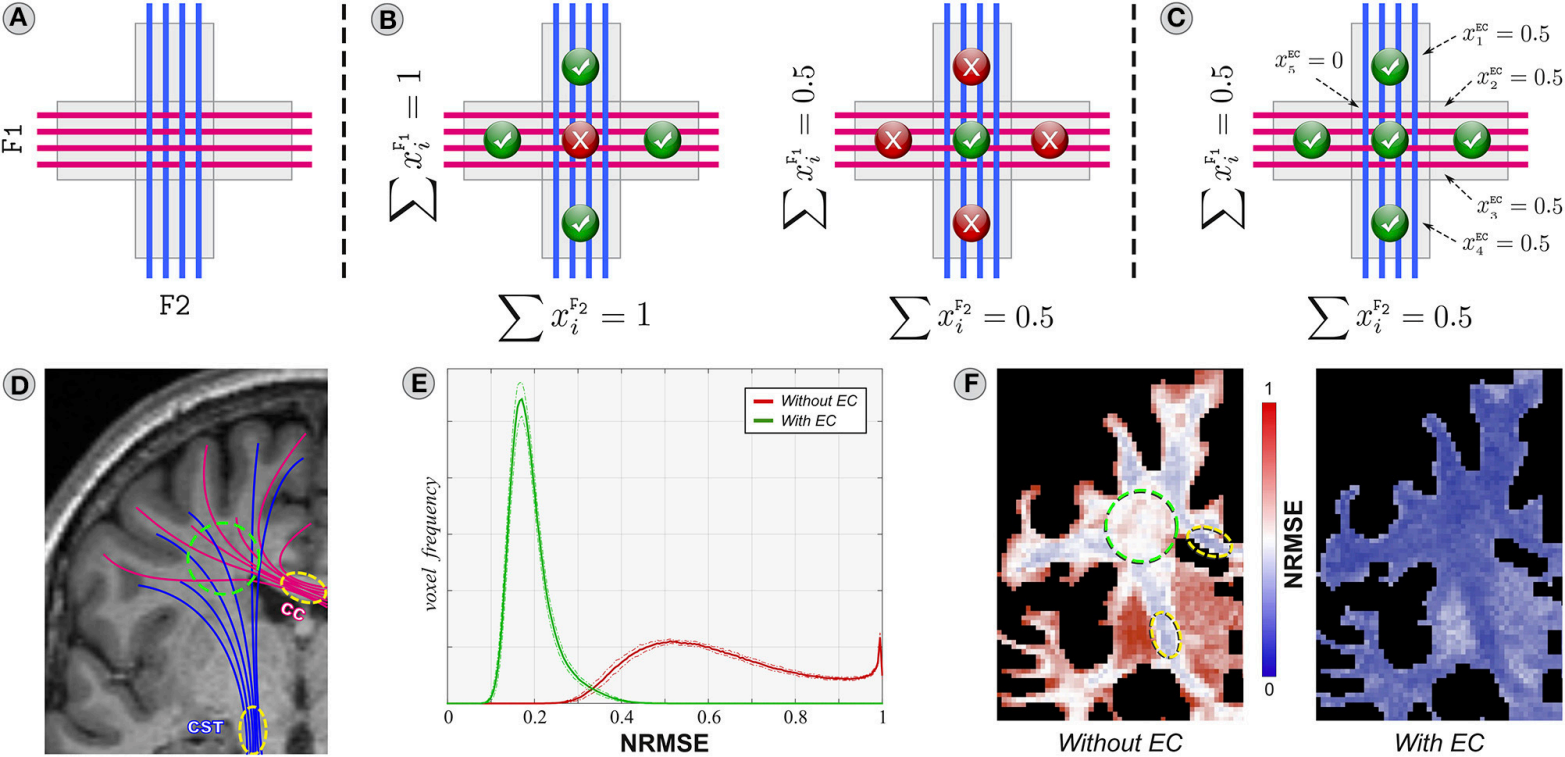
**Importance of the forward-model**. The toy problem in **(A)** is used to analyze the behavior of two different forward-models. The model in **(B)** assumes that the signal in each voxel originates exclusively from the tracts of the two fascicles F1 and F2 passing through it, whereas **(C)** considers all the possible water pools that can contribute to the dMRI signal, in particular the extra-cellular space (EC) around the axons. **(D96F)** Analogous situation in the brain using the HCP datasets. See description in the text for details.

Consider the *toy problem* in Figure 6A sketching a typical situation observed in the brain (Figure 6D): the corticospinal tract (CST) and the callosal projections of the corpus callosum (CC) are two major fascicles consisting of tightly-packed axons (yellow circles) that progressively fan out and eventually cross (green circle). As shown by several independent studies (Assaf and Basser, 2005; Alexander et al., 2010; Zhang et al., 2012), differences in the packing density are compensated by variations in the space around the axons themselves; note that this consideration is implicitly or explicitly assumed in all state-of-the-art microstructure techniques, and histological studies corroborate this hypothesis (Jespersen et al., 2010).

Let us first analyze a model in which the signal in a voxel is assumed to arise exclusively from the tracts passing through it, as e.g., in Reisert et al. (2011), Smith et al. (2013), and Pestilli et al. (2014). Therefore, given the tractogram in Figure 6A with the blue and magenta ideal fascicles of fibers *F1* and *F2*, their contribution to the image can be controlled via the unknowns $x_{1,\ldots ,4}^{F1}$ and $x_{1,\ldots ,4}^{F2}$. An assignment where $\overset{4}{\underset{i=1}{\sum }}x_{i}^{F1}=\overset{4}{\underset{i=1}{\sum }}x_{i}^{F2}=1$ (Figure 6B, left) would fit correctly the four corner voxels but in the central one the sum of the contributions would be over-estimated, i.e., $\sum x_{i}^{F1}+\sum x_{i}^{F2}=2$, violating the physical constraint that the volume fractions in a voxel intrinsically sum to one (Ramirez-Manzanares et al., 2007; Daducci et al., 2014b). Setting $\overset{4}{\underset{i=1}{\sum }}x_{i}^{F1}=\overset{4}{\underset{i=1}{\sum }}x_{i}^{F2}=0.5$ (Figure 6B, right) fixes this mismatch but then the signal in the four corners would be under-estimated. Clearly, there is a tension in the model and not all the voxels can be explained simultaneously; hence, a *suboptimal and biased solution* is always returned which, in turn, might affect the estimation of connectivity. The problem is that this model does not take into account that the dMRI signal originates from different water pools, i.e., intra- and extra-cellular, and that their contribution is not homogeneous (Assaf and Basser, 2005; Jespersen et al., 2010). In comparison, Figure 6C demonstrates that all the voxels can be accurately explained when the signal is modeled as a mixture of intra-cellular diffusion inside the tracts and extra-cellular diffusion around them, as e.g., in Sherbondy et al. (2010), Daducci et al. (2014a), and Reisert et al. (2014); in this work, this latter is assumed anisotropic and parallel to the axons, and its contribution in each voxel is controlled by the variables $x_{1,\ldots ,5}^{EC}$.

We also investigated how well the two models cope with such situations in *real brain* and we computed, for each HCP dataset, the normalized root-mean-square error (*NRMSE*) between the measured dMRI signal in each voxel and the one predicted from 500,000 tracts recovered with the iFOD2 algorithm; Figure 6E reports the overall mean (solid line) and standard deviation (dashed lines). Indeed, the histograms reveal rather high fitting errors (≈62% on average) if the extra-cellular space is not considered; in contrast, the fitting is significantly more accurate (≈19% error) when this compartment is included in the model. Figure 6F shows the results for a representative subject in a slice corresponding to panel d. In regions with densely-packed axons (yellow circles), where the extra-cellular space is modest, the fit is reasonably good (≈35%). However, large deviations from the measured signal can be observed in voxels with crossings (green circle, ≈60%) or partial volume with gray matter (≈90%), where indeed the (missing) extra-cellular compartment appears fundamental to properly explain all the voxel configurations present in the brain. The fitting error certainly provides us with useful information concerning how well a tractogram explains the measured data, but nothing about its biological plausibility, i.e., how well the tracts and the estimated contributions are in *agreement with the known brain anatomy*. To this aim, in the next section we will analyze the impact of these local fitting inaccuracies on the estimation of brain connectivity.

### 3.2. Biological plausibility

**Figure 7:**
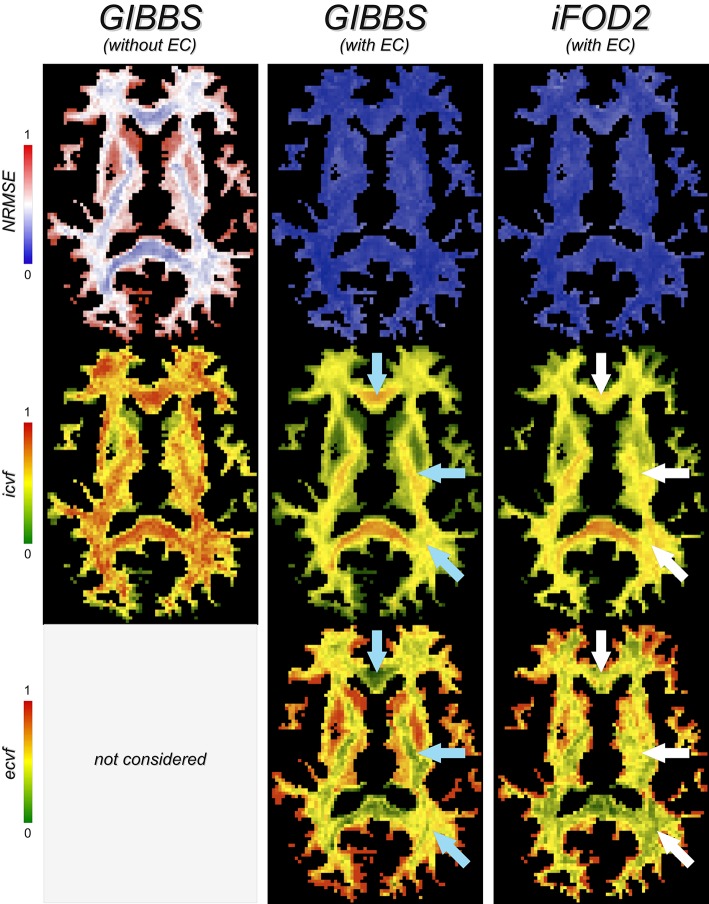
**Biological plausibility**. The fitting error (*NRMSE*) provides us with useful information about how well a tractogram explains the measured dMRI signal, but nothing concerning the biological plausibility of the estimated tract contributions. To this end, it can be very useful to inspect, for example, the fractions of the intra- (*icvf*) and extra-cellular (*ecvf*) compartments in each voxel as predicted by global optimization and compare to the known anatomy. For example, blue arrows highlight areas where the estimated fractions follow the expected pattern of neuronal density (see text for details), whereas white arrows point to the same regions where these contributions appear unrealistic in the last column.

Besides the fitting errors, Figure 7 inspects as well the fraction of the intra- (*icvf*) and extra-cellular (*ecvf*) compartments in each voxel as predicted with global optimization from the input tracts and using the two previous tissue-models. Tractography was performed on the two-shell dataset using both iFOD2 and GIBBS algorithms, without performing any filtering/pre-processing on the reconstructed tracts. The *NRMSE* maps confirm our earlier observation about the extra-cellular space, but now we can also evaluate its impact on the estimated parameters. Using the forward-model without extra-cellular compartment (left images), the *icvf* map shows indeed a spatial distribution that does not follow the expected pattern of neuronal density as found in previous studies (Assaf and Basser, 2005; Alexander et al., 2010; Jespersen et al., 2010; Zhang et al., 2012), that is higher density in the major white-matter bundles, e.g., CC and CST, homogeneous distribution in crossing regions and reduced values close to gray matter. In fact, the *icvf* map appears almost flat, *clear sign of an incorrect assessment of the tract contributions*. In contrast, when the extra-cellular space is considered in the forward-model (middle column), the estimated *icvf* and *ecvf* fractions closely resemble known anatomy (blue arrows); as expected, the *ecvf* map shows the opposite behavior. It is worth noting how this latter map actually follows the same spatial pattern as the fitting error of the model without extra-cellular space; we speculate it reflects, once more, the need to consider in the forward model all possible water pools that can contribute to the dMRI signal in order to correctly assess the actual contribution of the tracts. But this may not be enough. The rightmost maps show in fact that, despite the model being the same and the fitting accuracy very similar, distinct tractograms can lead to rather different spatial distributions of the estimated tract weights, which are not always consistent with the underlying anatomy (white arrows). Therefore, it is really *important not to rely only on the fitting errors to compare tractograms or evaluate a model*, but one should always have a look as well at the parameters of interest estimated with these microstructure informed tractography approaches, e.g., in this example the voxelwise *icvf* and *ecvf* maps computed from the tracts but, if more sophisticated biophysical models are used, also more biological indices of tissue microstructure.

## 4. Global-specific pitfalls

### 4.1. Signal intensity inhomogeneity

**Figure 8:**
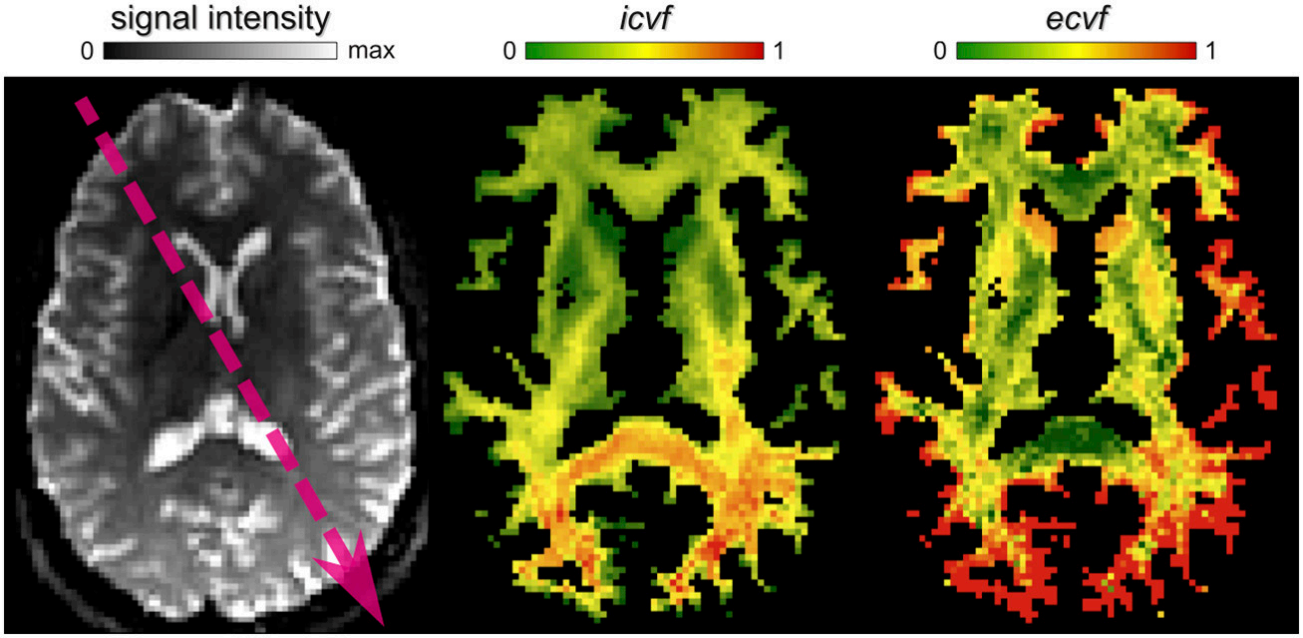
**Impact of signal inhomogeneities on global analyses**. In this example, the artificial modulation of the intensities, e.g., along the arrow direction, leads to a spatially-varying scaling of the estimated parameter maps that might be erroneously interpreted as a real change in the underlying connectivity.

Diffusion MR images can be affected by spatially-varying modulations of the signal intensity that are caused, besides actual tissue changes, by external factors such as local magnetic field variations, gradient nonlinearities or imperfections in the transmitter/receiver coils (Belaroussi et al., 2006). Although intensity inhomogeneities are usually not a problem for visual inspection of the images or voxelwise analyses, they can have severe consequences for methods considering all voxels in the optimization such as segmentation and registration (Vovk et al., 2007). Figure 8 shows an example when microstructure informed tractography is performed on raw dMRI images which are corrupted by such a bias field, whose main component can be observed in the direction pointed by the arrow. Compare these results, obtained from GIBBS, with the *icvf* and *ecvf* maps shown in Figure 7 (middle column), which were estimated after correcting this bias using the N4 algorithm (Tustison et al., 2010), as done in Daducci et al. (2014a) and Smith et al. (2015).

Without correction, this intensity modulation induces a scaling in the estimated parameters as well, because the actual contributions of the compartments are adjusted to fit the (scaled) measurements. An immediate consequence is that these artificial variations could be *mistakenly interpreted as real alterations in brain connectivity*, e.g., a decreased efficiency of communication in the frontal lobe in this example. But more subtle issues that are more difficult to spot can emerge. Current microstructure informed tractography techniques implicitly assume that the properties of the tracts, e.g., axonal volume, remain constant along their trajectories^3^. Although this assumption might be not totally correct, it is commonly accepted as good enough for current applications. In the presence of such bias, however, this constraint cannot be satisfied by the long association fibers connecting the frontal and occipital lobes, for instance; these tracts are likely to receive very low contributions as they are inconsistent with the (scaled) data and, as discussed in Section 2, artificial patterns might be introduced in the connectivity estimates. Please note that this observation applies to both top-down or bottom-up approaches. Hence, adequate procedures to correct/compensate for any signal inhomogeneity in the data are mandatory and should be employed in all analyses that use microstructure informed tractography techniques for assessing the connectivity.

### 4.2. Fiber length and convergence

**Figure 9:**
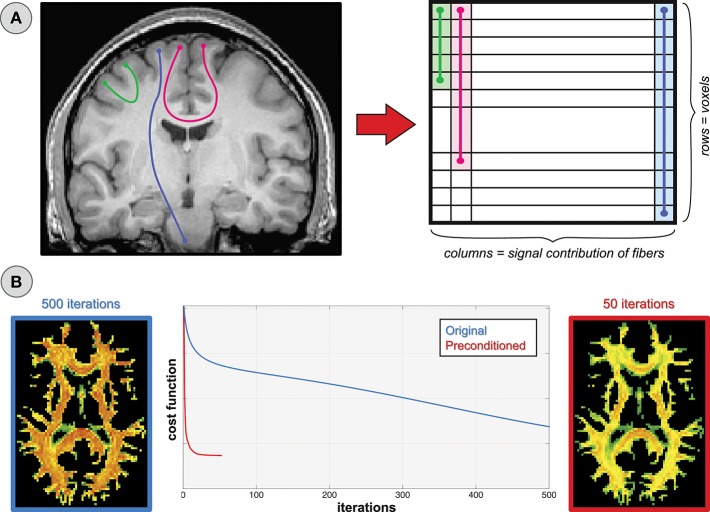
**Fiber length and convergence. (A)** The fibers in a tractogram vary greatly in length and thus their relative contributions to the dMRI image are quite different. **(B)** This imbalance may lead to very slow convergence and to a premature termination of the optimization process.

The approaches recently-proposed in Daducci et al. (2013), Daducci et al. (2014a), and Pestilli et al. (2014) have enabled a drastic reduction in the computation time for this class of problems by resorting to convex optimization. The convergence rate of classical iterative methods for solving large-scale problems of the form (2) largely depends on the spectral properties of the matrix **A**. Although this is usually not a concern in local reconstruction, for the reduced size of the problem and the availability of well-conditioned basis functions (Ramirez-Manzanares et al., 2007; Tournier et al., 2007), it can definitely be an issue for these dictionary-based algorithms. In fact, as illustrated in Figure 9A, axons vary greatly in length and, evidently, so do the tracts reconstructed with tractography. Their contribution to the image, and thus their relative importance in the optimization process, can differ quite considerably; this disparity can make the problem badly ill-conditioned and actions must be taken to reach convergence in a reasonable amount of time.

In Figure 9B, we monitor the progress of global reconstruction when the dictionary **A** is built from the original tractogram (blue line) and after normalizing its columns by their corresponding ℓ_2_-norms (red) so that all response functions are treated uniformly during optimization; in mathematics this operation is known as *preconditioning*. Although both problems will eventually converge to the same global minimum, in the preconditioned case the *icvf* map appears plausible already after 50 iterations, whereas the original problem is still far from an acceptable solution after 500. Apart from execution-time considerations, a slow convergence-rate may result in a premature termination of the optimization process, either because the maximum number of iterations is reached or the relative decrease in the cost function is very small and falls below a predefined threshold. Hence, the configuration returned at this stage might be sub-optimal if not arbitrarily flawed.

## 5. Discussion and conclusion

In this article, we have discussed the problem of *quantifying the structural connectivity in the brain* with diffusion MRI, placing a particular emphasis on the effectiveness of a novel class of algorithms known as microstructure informed tractography. In the spirit of *stimulating a constructive discussion in the field*, we have pointed out a series of pitfalls concerning the interpretation of the results from these methods so that these issues may be addressed. The intent of this work is not to suggest the idea that these novel techniques are flawed or to discourage their usage; on the contrary, we truly believe that *the proposed solutions are very promising* and represent a viable direction to further improve the estimation of brain connectivity. Nevertheless, the critical pitfalls as presented in this work indicate that, perhaps, these methods are *not yet ready for use in “real-world” applications*, due to a number of serious questions that remain unanswered. If these questions are overlooked or disregarded, a large potential for severely biased findings and erroneous conclusions exists.

To drive the discussion we have focused our analyses on dictionary-based methods (Daducci et al., 2013, 2014a; Pestilli et al., 2014), mainly because they provided us with (i) a solid mathematical framework and (ii) a convenient analogy with well-known local reconstruction techniques that allowed this discussion to remain concrete and easy to follow. However, it is worth noting that the concerns raised in this paper are quite fundamental and are relevant to any other global technique. For example, no matter what optimization strategy is used, all filtering approaches are unable to recover the false negative connections that are not included in the initial set of candidates. In a bottom-up algorithm, the potential tensions that a particular forward-model can introduce, as seen in Section 3.1, might drive the minimization procedure to suppress segments in some areas and prevent a number of streamlines to be eventually reconstructed. It is relatively straightforward to extend this list and extrapolate all the pitfalls previously described to other tractography techniques; however, considering the variety of algorithms available in the literature, such description would be too lengthy for the scope of this work. To summarize our general observations, in the following we outline a list of the main *take-home messages and open challenges* that potential users must keep in mind when trying to use microstructure informed tractography techniques in their studies.

- As the methods covered in this work are all based on global optimization, i.e., all voxels considered simultaneously, it is important to realize that a problem in a specific location or fiber bundle may have side effects anywhere else in the brain. For example, if a fiber bundle is not reconstructed, some other tracts in the tractogram might be assigned contributions to compensate and fit the measured data. Conversely, if some tracts are recovered multiple times, e.g., because easy to track with tractography, then their weights may be arbitrarily split among the duplicates. As a consequence, spurious tracts might be detected as plausible and genuine ones as invalid. Therefore, to make valid inferences about the data with these tools, the composition as well as the features of the tractogram are absolutely critical.
- A large number of models has been developed to estimate properties of the neuronal tissue in a voxel from the observed data, but there is still considerable debate about which one provides the most accurate and useful features. Moving the fitting from a single voxel to the whole brain does not solve this dilemma but, instead, it may further amplify the uncertainty. Different models explain the data more or less well and may result in rather contrasting feature estimates; inaccuracies in a voxel, combined with the global nature of the fitting problem, can lead to unpredictable results occurring in any other location of the brain or lead to biologically unfeasible solutions. Carefully ponder the choice of the tissue model used to assess the contribution of the tracts and consider its impact in the interpretation of the observed outcomes regarding the tractogram.
- The fitting error is certainly the first quantity one inspects to evaluate the goodness-of-fit of a given model. Indeed, high errors are a good indicator that the model fits the data poorly and the estimated parameters are most likely unreliable. However, the other way around is not always true. These global methods optimize over an extremely high number of unknowns and the degrees-of-freedom of such problems are very large; hence, overfitting can easily occur and the estimated parameters may not reflect the underlying anatomy. Therefore, it is utterly important to inspect and carefully examine as well the maps of the obtained parameters in order to actually evaluate the biological validity of the tractogram at hand.
- Most existing tractography algorithms do not enforce in the tracking process the anatomical prior that fibers must origin and terminate in the gray matter; as a consequence, a lot of recovered tracts actually stop prematurely in the white matter. Although this is not an issue when visually inspecting tractograms, these partial fibers can severely bias the quantification of the structural connectivity and their careful handling is essential for unbiased estimations.
- Due to a number of acquisition artifacts, MR images are usually affected by spatial variations of the signal intensity. This modulation may introduce artificial patterns in the estimated parameters which, as previously pointed out, can occur rather far from the actual location of the artifact in the image. In the case of analyses with diffusion MRI, these variations might be mistakenly interpreted as real alterations in the connectivity; hence, correction strategies to remove these artifacts from the images before applying these global techniques are crucial, otherwise unpredictable results might be obtained.

That being said, we encourage researchers working on brain connectivity with diffusion MRI to try these tools and explore the new and exciting possibilities they offer, but also be aware of the current limitations. By acknowledging their shortcomings as previously discussed we hope to *stimulate new ideas and research in this direction*, paving the way to enable, in the near future, truly quantitative and biologically meaningful analyses of brain connectivity.

## Author contributions

Study conception and design: AD, ADP, JT; analysis and interpretation of experimental results: AD, ADP, MD; drafting of manuscript: AD, MD; critical revision: all authors.

### Conflict of interest statement

The authors declare that the research was conducted in the absence of any commercial or financial relationships that could be construed as a potential conflict of interest.

## Footnotes

^1^A number of open-source software packages are publicly available, e.g., https://github.com/MRtrix3/, https://github.com/daducci/COMMIT/ and https://github.com/francopestilli/life/. These tools are well documented and also contain demos/tutorials to guide users from the raw data to the processed results.

^2^https://github.com/daducci/COMMIT.

^3^One could argue that almost all tractography methods actually use this assumption. To date the sole exception is represented by MesoFT (Reisert et al., 2014), which allows for spatially-varying response functions but still enforces the estimated parameters to be similar in adjacent segments of the tracts.
